# HIV-1 Nef Protein Structures Associated with Brain Infection and Dementia Pathogenesis

**DOI:** 10.1371/journal.pone.0016659

**Published:** 2011-02-09

**Authors:** Susanna L. Lamers, Art F. Y. Poon, Michael S. McGrath

**Affiliations:** 1 Polytechnic Institute of New York University, New York, New York, United States of America; 2 BioInfoExperts, Thibodaux, Louisiana, United States of America; 3 British Columbia Centre for HIV/AIDs Excellence, British Columbia, Canada; 4 Department of Laboratory Medicine, University of California San Francisco, San Francisco, California, United States of America; 5 The AIDS and Cancer Specimen Resource (West Coast ACSR), San Francisco, California, United States of America; The Centre for Research and Technology, Hellas, Greece

## Abstract

The difference between regional rates of HIV-associated dementia (HAD) in patients infected with different subtypes of HIV suggests that genetic determinants exist within HIV that influence the ability of the virus to replicate in the central nervous system (in Uganda, Africa, subtype D HAD rate is 89%, while subtype A HAD rate is 24%). HIV-1 nef is a multifunctional protein with known toxic effects in the brain compartment. The goal of the current study was to identify if specific three-dimensional nef structures may be linked to patients who developed HAD. HIV-1 nef structures were computationally derived for consensus brain and non-brain sequences from a panel of patients infected with subtype B who died due to varied disease pathologies and consensus subtype A and subtype D sequences from Uganda. Site directed mutation analysis identified signatures in brain structures that appear to change binding potentials and could affect folding conformations of brain-associated structures. Despite the large sequence variation between HIV subtypes, structural alignments confirmed that viral structures derived from patients with HAD were more similar to subtype D structures than to structures derived from patient sequences without HAD. Furthermore, structures derived from brain sequences of patients with HAD were more similar to subtype D structures than they were to their own non-brain structures. The potential finding of a brain-specific nef structure indicates that HAD may result from genetic alterations that alter the folding or binding potential of the protein.

## Introduction

The ability to identify neurotropic strains of the Human Immunodeficiency Virus type 1 (HIV-1) is relevant to many different fields of HIV research. This is reflected in numerous research articles that attempt to 1) explain viral evolution in the central nervous system (CNS), 2) identify HIV-1 genotypes associated with CNS viral compartmentalization, 3) assess molecular diversity of various HIV-1 proteins and their functional consequences in the CNS, and 4) develop treatments for individuals suffering from the effects of HIV-associated dementia (HAD). The CNS is a viral reservoir in which the primary HIV-infected cell-type is the macrophage, a cell-type that is not targeted by current antiretroviral therapies. HAD develops when HIV-infected monocytes or macrophages can pass into the brain and infect resident brain macrophages; next, infected macrophages then release cytokines that attract more macrophages to sites of infection and a self-inflammatory process transpires [1], [2]. At autopsy, patients with HAD display an abundance of HIV-infected macrophages deep within brain tissues, particularly in the frontal and temporal lobes. It is interesting that in some patients without any trace of clinical HAD pathology, HIV DNA exists within brain tissues. On the other hand, other HIV-infected patients have no detectable HIV DNA in the brain at death [3], [4]. These findings suggest that some HIV variants may be more capable of entering the CNS, but are less pathogenic in the brain environment, whereas other HIV variants might be more efficient in both brain infiltration and in setting up the HAD self-inflammatory macrophage environment. Another explanation for the finding of HIV in the brain without HAD development, could be late-stage HIV infiltration, which could arise from atherosclerosis or breakdown of the blood brain barrier near death [3], [5], [6].

HIV is classified into numerous subtypes based on genetic similarities. In 1999 Kanki et al. found that subtype A has a longer AIDS-free survival than non-A (Kanki et al, 1999). Other studies showed that subtype D has a shorter time to death and AIDS than subtype A, C, or recombinant forms (Vasan et al, 2006); and in subtype C, CCR5/non–syncytia-forming viruses are almost always the dominant form even in late stages of infection (Tscherning et al, 1998). In 2009, Sacktor et al. demonstrated that individuals in a localized region of Uganda, Africa infected with subtype D HIV-1 had an 89% chance of developing HAD, while those infected with subtype A had only a 24% chance of developing HAD [7]. A report from Ethiopia suggested that HAD is infrequent in clade C (Clifford et al, 2007). A review by Liner et al. also pointed to an association between HIV subtypes and neurological disease [8]. We recently reported that the HIV-1 nef protein evolves differently in brain tissues from patients who develop HAD than from patients who do not [3]. Given these observations, a viral genetic link to HAD is possible; however, due to the extensive diversity in the HIV genome within an individual and especially between HIV-1 subtypes, the ability to identify a HAD-specific virus based on DNA sequence data alone does not exist.

Several HIV proteins have been implicated in the development of HAD, in particular nef, tat, env, vpu and the LTR [9], [10], [11], [12], [13], [14]. HIV-1 nef is a multi-factorial protein required to maintain high viral loads [15]. The loss of nef function results in delayed or absent AIDS progression [16], [17], [18]. It is the first protein to accumulate following infection of macrophages and is estimated to represent three-quarters of the viral load in the infected cell [19]. Additionally, nef promotes the survival of infected cells [20] and migration [21]. In the cell, nef can bind MHC class II receptors, allowing it to interfere with a variety of cellular process such as triggering rapid CD4 endocytosis, targeting the trans-golgi network, activating phosphatidylinositol 3-kinases and lysosomal degredation [22], [23]. Several studies have showed that exposure of neuronal cell cultures to nef resulted in neurotoxicity [24], [25], [26], [27]. Within the brain of HIV-1-infected patients, nef is found in both macrophages and astrocytes [28]. A study by Ranki et al. found that astrocyte infection is associated with moderate to severe dementia; additionally, the study found that astrocytes preferentially express HIV nef and rev proteins and that six out of seven patients with nef-positive astrocytes suffered from moderate to severe dementia [29]. Other studies indicated that stable expression of nef may alter growth properties of human astrocytes [28] and that nef/LTR deleted viruses have either diminished neurotropism or insufficient systemic viral replication for entry into the CNS [30]. Olivieri et al. performed a genetic analysis of nef that demonstrated the existence of adaptive selection in the brain, thus suggesting altered requirements for efficient replication of nef in macrophages or when exposed to brain-specific immune selection pressures [31].

Considering the large body of work that has potentially linked the HIV nef protein to HAD development, defined the effects of nef and analyzed how nef amino acid variation may contribute to its structural properties, our goal was to determine if clues to dementia pathogenesis could be discerned from the three-dimensional (3D) structure of the nef protein derived from patients with and without HAD. We utilized 1153 sequences to generate consensus sequences for brain and non-brain tissues from HIV-1 infected patients, including subtype A and D sequences from Uganda. These consensus sequences were then used to computationally generate protein structures. Keeping in mind that the nef protein is flexible in its natural environment, a snapshot of the protein calculated in an identical environment from a cohort of individuals displaying different disease pathogenesis could elucidate structure-function similarities that are not discernible by viewing sequence information alone.

## Methods

### Samples

Frozen autopsy tissues from patients and accompanying pathology records were obtained from the University of California at San Francisco AIDS and Cancer Specimen Resource (ACSR) (http://acsr.ucsf.edu). The tissues were obtained from patients after appropriate written consent and after a de-identification procedure was applied. Patient designations used throughout this study were generated randomly as shorthand used by technicians who performed the studies and do not correlate to patient information. The ACSR is recognized by the Office of Biorepositories and Biospecimen Research at the National Institutes of Health as being HIPAA compliant and in accordance with the ethical standards of the Declaration of Helsinki. Additionally, all material was obtained under written approval from the UCSF committee on human research. The UCSF committee on human research approved the study.

### Patients and tissues

Extensive details concerning patient tissue samples utilized in the current study have been published previously [32]. Briefly, patients AM and IV developed metastatic AIDS-related lymphoma (ARL), patients GA and CX developed progressive HAD, patient BW developed ARL, including meningial lymphoma, patient DY had suffered from recurring *Mycobacterium avium* complex infection (MAC) for years and developed an aggressive form of HAD in the weeks prior to death.

### Quantitative PCR

A quantitative polymerase chain reaction (QPCR) was used to identify if HIV DNA was present in significant amounts in the brain tissues from each patient. First, 50 ng of genomic DNA was prepared by QIAamp DNA Mini Kit (QIAGEN company, Valencia, CA) and quantified using ultra-violet spectrometry at an absorbance of 260 nm (Nano-Drop® ND-1000 UV-Vis Spectrophotometer, NanoDropTechnologies, Wilmington, Delaware). Next, HIV copy number/genomic equivalent within each tissue was calculated by QPCR using primers SK38 (5′-*ATA ATC CAC CTA TCC CAG TAG GAG AAA T*-3′) and SK39 (5′-*TTT GGT CCT TGT CTT ATG TCC AGA ATG C*-3′) yielding a 110 bp fragment of the gag sequence. The single-copy human c-jun gene was used as genomic equivalent standard for quantitation purposes. The amplification protocol comprised one cycle denaturation at 95°C for 10 minutes, followed by 30 cycles of 95°C for 15 s, 60°C for 5 s, and 72°C for 12 s (Roche's FastStart NA Master SYBR Green 1 Kit, Roche Applied Science, Indianapolis, IN). 1 HIV copy number/5000 cell equivalents was used as a cut-off, with all values higher considered HIV-1 positive. This ratio had replicates within 15% coefficient of variation; a greater amount was associated with intra-assay noise. This approach indicated that four of the six patients had QPCR amplifiable amounts of HIV in the brain at the time of death (GA, CX, BW and DY).

### Sequences

**Figure 1:**
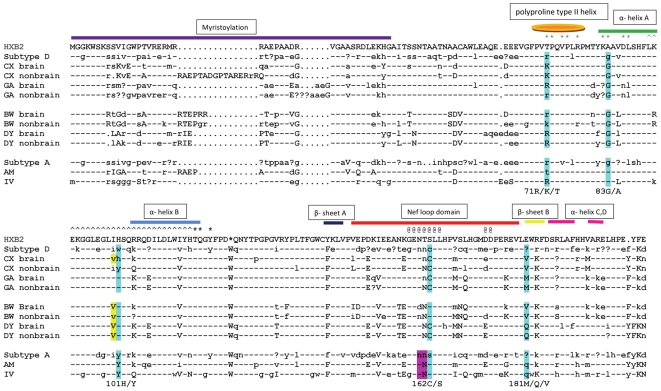
Consensus Sequence Alignment. Consensus sequences were constructed using the program Seqpublish for sequences derived for all patients. For the patients with brain infection, consensus sequences were generated independently for brain and non-brain sequences. Capitol letters indicate positions conserved at 95%, lowercase letters are positions conserved at 50–95% and a question mark indicates non-conserved positions. Dashes indicate identity to HIV clone HXB2. Periods indicate gapped positions. The blue highlighted positions correspond to mutable amino positions potentially associated with disease pathology as calculated with SDM. The yellow highlighted position, immediately prior to position 101 is necessary for the mutation H to Y to be significantly destabilizing. The purple highlighted positions show that a glycosylation motif exists in all non-brain structures that may be used for structural, rather than functional purposes. Further information on these positions can be found in Table 1. Secondary structure information is shown above the sequence. Asterisks (*) indicate positions necessary for SH3 binding. The carrots (∧) indicate the domain associated with chemokines binding. The At symbols (@) indicate a dileucine binding motif associated with AP-2 binding.

Amplification, cloning, and sequencing of HIV DNA were performed as follows. Genomic DNA was extracted from 10–30 mg of each tissue using the QIAmp DNA Mini Kit from Qiagen according to the manufacturer's instructions. A 3.3 kb HIV fragment, extending from the 5′ env to the 3′LTR U3 domain was amplified by PCR using the primers EnvF1 (*AAC ATG TGG AAA AAT AAC ATG GT*) and NefR1 (*ACT TDA AGC ACT CAA GGC AA*) under the following conditions: an initial denaturation step of 94°C for 5 min followed by 35 cycles of 94°C for 30 sec, 55°C for 30 sec, 68°C for 3 min 20 sec, and a final extension at 68°C for 8 min, in 50 µl volume using 600 ng of template DNA, 10 µl of 10× buffer (Invitrogen), 10 mM deoxynucleoside triphosphates (Invitrogen) 60 µM of each primer, and 2.5 units of Invitrogen Platinum-Taq polymerase. Products were cloned into the pCR2.1-TOPO vector according to the manufacturer's instructions and the resultant colonies were screened for the proper insert using the PCR protocol described above. Sequencing was performed by ELIM Biopharmaceuticals, Hayward, CA. For each patient five to seven tissues were studied. Approximately 20–30 sequences were derived from each tissue (Table 1, Genbank accession numbers GQ868779-GQ869380). In addition, all nef subtype D (67 sequences) and subtype A (55 sequences) sequences derived from patients in Uganda, Africa were downloaded from the public domain HIV Sequence Data Base at the Los Alamos Laboratories (www.hiv.lanl.gov). Sequences were aligned using Clustal with the MEGA 4.0 software package (http://www.megasoftware.net/) and a phylogenetic tree using all protein sequences was calculated using the neighbor-joining method and the PAM matrix (Figure S1) and a bootstrap value of 500 replicate samplings. Protein sequences were used in the analysis so that only nonsynonymous DNA mutations would be observed in the tree and provide a view of the evolutionary distance between patients and subtypes at the functional level. Phylogenies were visualized using Figtree (http://tree.bio.ed.ac.uk/software/figtree/). Next, a consensus nef sequence was generated for brain and non-brain sequences for each patient and subtype A and D populations using the program Seqpublish at the Los Alamos HIV Data Base (www.hiv.lanl.gov). Consensus sequences were aligned to the molecular clone HXB2 (Figure 1).

**Table 1:** Sequences used for consensus sequence building.

Sequence Population	Number of Tissues	Number of sequences
CX brain	7	147
CX non-brain	2	49
GA brain	3	79
GA non-brain	2	50
BW brain	5	119
BW non-brain	1	36
DY brain	5	103
DY non-brain	2	49
AM non-brain	10	231
IV non-brain	10	168
Subtype D	1	67
Subtype A	1	55

### Calculation of disease-associated point mutations

Potential disease-associated point mutations were calculated using the Site Directed Mutator (SDM, http://mordred.bioc.cam.ac.uk/~sdm/sdm.php). SDM compares structures in order to determine if point mutations would significantly modify the structure using a statistical potential energy function developed by Topham et al. [33]. SDM has been successfully used to identify disease-associated modifications within protein structures [34]. The program calculates the ΔΔG, which is the difference in free energy in the unfolding of the changed vs. unchanged positions within structures. A value less than -1.0 indicates that an amino acid substitution significantly modifies the structure and would unfold at lower temperature.

### Generation of nef 3D structures

A structural model of the complete nef protein was assembled from partial structures published in the Research Collaboratory for Structural Bioinformatics (RCSB) Protein Data Bank (PDB) as follows: a core domain structure spanning residues 56–159 and 175–206 (numbering relative to the HIV reference sequence HXB2; RCSB PDB accession number 2NEF]); and an anchor domain structure spanning residues 2–57 (RCSB PDB accession number 1QA5). The core and anchor domain were merged using Swiss-PDB Viewer and local energy minimization was performed using UCSF Chimera (http://www.cgl.ucsf.edu/chimera/). The composite structure was further modified using the same methods for the insertion of 15 residues to restore the disordered loop bounded by core residues E and D and performing local energy minimization of this loop. Further energy minimization was carried out using GROMACS (http://www.gromacs.org). We hypothetically ‘immersed’ the molecule in an aqueous solution bounded by a cubic box, and performed energy minimization of this system using a simulated annealing method (with annealing temperatures 425K, 375K, 325K, 275K, 245K, and 203K scheduled at time points 0, 100, 200, 300, 400, and 500 picoseconds, respectively) to a force tolerance of 10∧-3 kJ mol nm. Lacking topological information about myristol, it was necessary to omit the myristolated N-terminal methionine residue from the model. Next, we replaced six water molecules with sodium ions in order to neutralize the net negative charge of the system and repeated the energy minimization step. By modifying residues in the Swiss-PDB Viewer and performing energy minimization in GROMACS, we generated patient structures from the base structure by varying the number of solvent molecules replaced by charged ions as necessary to neutralize the system.

### Visualization and alignment of structures

**Figure 2:**
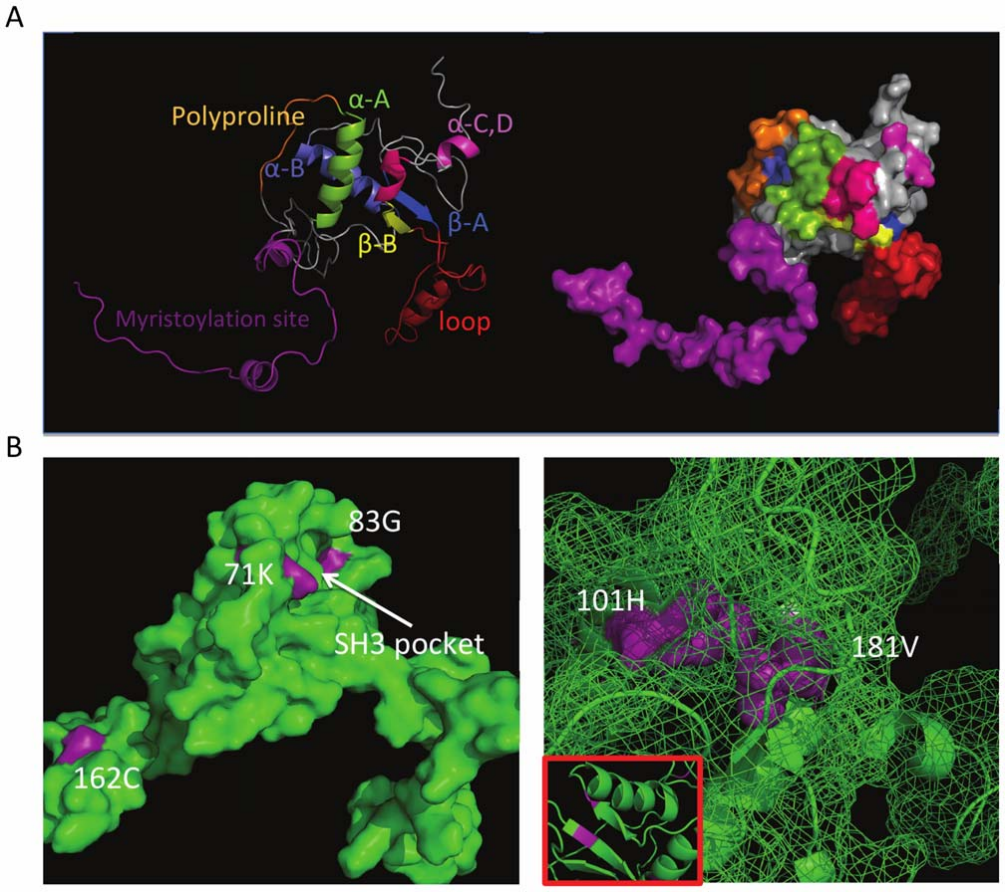
3D HIV-1 nef tertiary structure (Panel A) and potential disease-associated mutations (Panels B and C). In Panel A, the colors correspond to the secondary structure shown above the alignment in Figure 1. A cartoon format is shown on the left and the structure displayed as the surface that would be traced out by water in contact with the protein at all possible positions on the right. Panels B and C show different views of the nef protein. Disease associated mutations are shown in purple and are numbered according to Table 1. Panel A shows a surface view of the protein with positions 162, 71, and 83 highlighted and identifies the location of the SH3 pocket. Panel B shows a mesh view, with the cartoon structure superimposed; the inset boxed in red shows the relationship of positions 101 and 181 in the 3D structure, which appear on opposite strands of the beta sheet formation.

**Figure 3:**
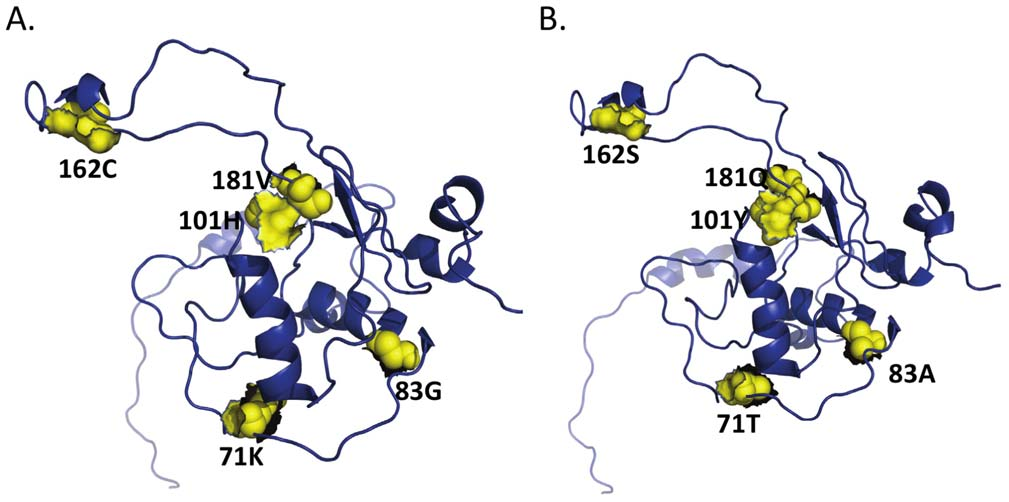
Representative brain and non-brain structures. Structures derived from patient CX brain consensus sequence (Panel A) and patient AM consensus sequence (Panel B) are shown for comparison of brain and non-brain structure. Signature positions are yellow, labeled according to the alignment in Figure 1 and shown as spheres with the surface superimposed.

The program PyMOL (http://www.pymol.org/) was used to visualize and color structures as shown in Figure 2 and Figure 3.

### Distance analysis

**Figure 4:**
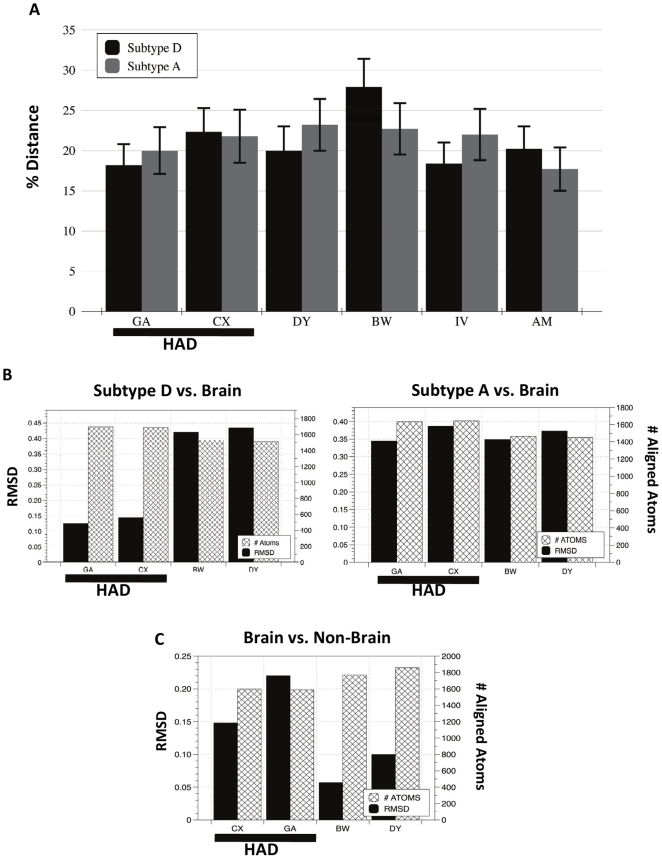
Variation among sequence populations and consensus structures. In panel A, the distance between patient sequences was compared to subtype D (light blue bars) and subtype A (dark blue bars) using Mega 4.0. Standard error bars are shown in red. Panel B displays the RMSD values for consensus patient brain structures aligned to consensus subtype D and consensus subtype A structures. Patient IDs are on the X-axis. Each graph provides the RMSD (left y-axis) and number of atoms compared (right y-axis) in each structural alignment. In Panel C RMSD values for the comparison of brain vs. non-brain structures within each patient are provided. Patient IDs are on the x-axis.

The variation between each patient sequence population to subtype A and subtype D populations were calculated using MEGA 4.0 (Figure 4A) and the Tamura 3-parameter molecular model using 1000 bootstrap replicates.

### Structural variation

**Figure 5:**
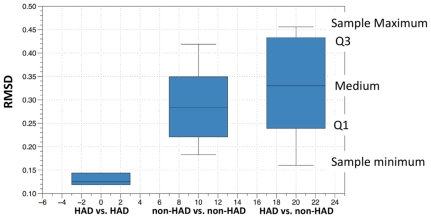
Box and Whisker plot comparing RMSD values from three populations of structures. The diagram describes the two groups of data through six statistical summaries: 1) the sample minimum, 2) lower quartile (Q1), 3) median, (4) upper quartile (Q3), 5) sample maximum and 6) outliers. The spaces between the different parts of the box indicate the spread of the data.

The deviation between consensus 3D protein structures was calculated using PyMOL. PyMOL aligns by atomic coordinates and calculates the minimum root square mean deviation (RMSD) between aligned atoms. The RMSD is a well-known standard for measuring structural similarity between two structures. Since PyMOL will not align highly divergent residues, the number of aligned atoms used to calculate the RMSD for each alignment was obtained to determine if a similar number of atoms were aligned in each comparison (Figure 4B). A box and whisker plot summarizing the comparison of all patients' structures to each other (37 structural comparisons) was generated (Figure 5). In the first plot in Figure 5 (HAD vs. HAD), the range of the RMSD values for comparing subtype D, CX and GA is shown. In the second plot (non-HAD vs. non-HAD), the range of the RMSD values for comparing structures subtype A, BW, DY, AM and IV is shown. In the third plot, the range of the RMSD values for all structural comparisons is shown. All graphs were generated using the program DataGraph version 2.1.1 (http://www.visualdatatools.com/DataGraph/).

## Results

### Phylogenetic Analysis

Phylogenetic analysis of all data used in the study showed that the sequences from each patient were unique to that patient and clustered in individual clades on the tree (Figure S1). In addition, subtype A and D sequences formed distinct clades on the tree that were unrelated to patient sequence populations. Phylogenetic analysis of nef nucleotide sequences for each patient has been previously published and showed that there were some significant subpopulations within patient datasets, especially between brain and non-brain sequences for patients GA and CX [3].

### Consensus Sequence Alignment and Mutational Analysis

For the purpose of discussion, the term “brain sequences/structures” refers to sequences from patients who had HIV within the brain at the time of death and no signs of progressive HAD (BW, DY), “HAD sequences/structures” refers to patients who died due to long-term progressive HAD (GA, CX), and “non-HAD sequences/structures” refers to patients who did not have amplifiable amounts of HIV within the brain at the time of death and showed no sign of brain disease (AM, IV). Several positions along the alignment appeared related to brain, HAD and non-HAD sequences. Using the program SDM, we identified the mutations that would significantly alter the Gibbs Free Energy (ΔG); the positions are highlighted in the alignment shown in Figure 1. The changes in ΔG for the non-HAD vs. HAD are shown in Table 2.

**Table 2:** Potential disease-associated mutations in HIV nef.

Position	Mutation	ΔΔG^1^	Alter Structure?
71	R/T	−2.727	Yes
71	K/T	−1.003	No
71	R/K	−0.302	No
83	A/G	−2.851	Yes
101	H/Y^2^	−3.864	Yes
162	S/C	−5.587	Yes
181	M/Q	−2.465	Yes
181	V/Q	−2.834	Yes
181	**M/V**	−0.646	No

Positions 83, 101, and 162 displayed strong signatures among the groups. At position 83, all of the HAD and brain patients contained a glycine (G), whereas all non-HAD patients contained an alanine (A). At position 101, all non-HAD sequences contained a tyrosine (Y), all brain sequences contained a histidine (H), and most HAD sequences also contained an H. An interesting disease-associated position was found at position 162, which occurs in the center of the nef loop domain; all of the HAD patients and the subtype D sequence contained a Cysteine (C), one of the brain patients contained a C and none of the non-brain sequences contained a C in this position. More interesting is that a C substitution results in the elimination of a glycosylation motif (NXT or NXS, where X is any amino acid except P). Although nef is not glycosylated during HIV infection, the substitution introduces a residue that is free to interact by di-sulfide bonding with cysteines within the protein or with other proteins in the environment. Patient BW did not have a C in this position; however, he did not have a glycosylation motif either. In patient BW brain structures, the ΔΔG of a mutation to a C is -7.359, much higher than the non-brain patients. At position 181 the mutation of a valine (V) or methionine (M) to a glutamine (Q) is destabilizing. All of HAD sequences had a V or an M in this position and all of the non-HAD sequences contained a Q. Brain sequences were again mixed with one patient containing the V residue and one patient containing the Q residue.

Although some signature positions displayed a clear separation among the three groups, some were less convincing, yet still notable. In patient GA at position 71 there was a mixture of R and lysine (K) in HAD and brain sequences. In the same position, one non-brain patient contained all R while the other contained a mixture of threonine (T), K and R. Although the change from and R or K to a T is considered destabilizing, the signature in our study population is only weakly associated to the brain or HAD phenotype. Primarily the occurrence of a T in this position in any sequence is interesting because it is necessary for SH3 binding.

### HIV-1 nef structural analysis

Figure 2A shows the structure of the complete nef protein obtained by combining PDB structures 2NEF (transmembrane domain) and 1QA5 (myristoylation domain). On the left is the backbone representation and on the right is the surface representation with the same color scheme. The figure shows that much of the tertiary structure is exposed to the surface of the protein with particular exposure of the nef loop domain (red). In Figure 2B (left panel), the potential signature amino acids 83, 71, and 162 are seen to some extent on the surface of the molecule. Positions 71 and 83 are shown within the well-characterized SH3 binding pocket of nef [35]. In Figure 2B (right panel), the structure is shown as a mesh so that it is possible to visualize through the structure and observe the close proximity of positions 181 and 101. These positions are directly adjacent to each other in the hydrophobic core structure of the internal anti-parallel beta sheet of nef (Figure 2B, right panel). Considering that both of these mutations can alter the structure, this finding may indicate that the sites co-evolve to maintain a specific protein conformation. Figure 3 shows a comparison of brain structure derived from patient CX consensus sequence to a non-brain structure derived from patient AM's consensus sequence. The rotation of the structure is different from that in Figure 2 so that all signature positions (yellow spheres), and their relationship to each other can be seen clearly.

### Distance analysis

The distance between patient subtype B sequence populations from subtype D and A populations was assessed to determine if there was any significant difference between the populations at the amino acid level (Figure 4A). Overall, no significant genetic difference at the nucleic acid level between subtype A and D to patient sequence populations was found. The establishment of no significant genetic distance between subtype B to subtype D and A sequence populations is important when considering the results of structural alignments in the following section.

### Structure alignments

Figure 4B shows the RMSD and number of atoms aligned in the comparison of patients' structures to subtype A and D structures. Overall, the number of atoms aligned (hashed bars) in each analysis did not vary significantly (p = >0.0001), thus indicating that the alignments across various structures were of equal integrity. In Figure 4B (left panel), HAD structures are significantly more related to the subtype D structure than non-HAD structures are related to the subtype D structure (p = >0.05). In addition, a comparison of Figure 4B (left panel) and 4B (right panel) indicates nef structures derived from patient's brain tissues with HAD were significantly more similar (p = >0.01) to the consensus subtype D “dementia” nef structure (RSMD  = 0.13) than to the consensus subtype A “non-dementia” nef structure (RSMD = 0.34). There was no statistical difference between non-HAD structures aligned to either subtype D or A structures (p = 1.0). An alignment of patient CX and GA brain structures also had a very low RMSD value of 0.11 (data not shown in Figure 4). In Figure 4C we compared the brain to non-brain structures within each patient to assess the within-patient structural variation. Interestingly, the two HAD patients varied almost two-fold between their brain and non-brain structures than the non-HAD patients (BW and DY). Furthermore, CX brain structure is slightly closer to the subtype D structure (RMSD  = 0.15) than to his non-brain structure (RMSD  = 0.14) and GA has a large increase in distance between his brain and non-brain structure (RMSD  = 0.22) as compared to his brain and subtype D structure (RMSD  = 0.13). Overall, the results from structural alignments showed that HAD derived structures from the brain were more related to subtype D structures than the non-HAD structures. This result is quite interesting considering that at the genetic level, no significant difference between HAD and non-HAD sequences to subtype A or D was found.

In Figure 5, 3D structures from all patients were aligned to each other resulting in 37 RMSD values that describe the overall variation between structures in patients with varied disease pathologies. The results were plotted in a box and whisker plot and the results indicated that HAD structures are much more similar to each other than non-HAD structures are similar to each other. In addition, the results show that when HAD structures are compared to non-HAD structures, an even larger spread of data occurs.

## Discussion

In this study we computationally generated and compared HIV nef structures from patients who died due to different primary HIV-associated pathologies. In particular, we were interested in two patients who died due to HAD and questioned if they harbored unique HIV nef structures within the macrophage-associated tissues of the brain. In an earlier study, we noted that many HIV-infected patients did not have significant amounts of HIV within brain tissues at death [4]. Other patients have HIV within the brain, but do not develop symptoms related to HAD. Because of the multiple functions associated with the HIV nef protein in the brain: 1) neuronal degeneration [36], 2) the production of cellular cytokines [37] and, 3) the potential interference of cellular pathways involved in central nervous system integrity [10], we utilized the nef sequence in the current analysis. HIV-1 nef modulates signaling in infected and bystander macrophages, thereby facilitating disease progression. Geyer et al. and Gerlach et al. made several important findings concerning the structure of the nef protein: 1) the structure of nef is somewhat flexible, with several well-defined regions that control many of its functions, including a myristoylation domain that allows the protein to anchor to cellular membranes, a loop within the core domain designated for signaling and structural integrity and a second flexible loop for trafficking and internalization, 2) flexible regions form large surfaces of the protein that are readily accessible for interactions and are capable of undergoing important conformational changes and 3) the less-structured nature of the flexible regions correlates with a higher degree of sequence variability [38], [39].

A number of cellular functions associated with macrophages have been assigned to the nef protein, including inhibition of apoptosis, sustained viral transcription, production of pro-inflammatory cytokines and chemokines and expression of tumor necrosis factor (TNF) on macrophages [40]. Costa et al. found a nef consensus motif that binds the actin cortical patch component (AIP1) *in vitro* and *in vivo* [41]. AIP1 is a critical intermediate in the formation of multivesicular bodies (MVBs) that play an important role in the budding and release of viruses from infected cells. This function of nef was reproduced in primary macrophages, where nef proteins led to increased release of new viral particles from infected cells [41]. In addition, HIV-1 nef binds to the macrophage-specific Src tyrosine family member Hck through its SH3 domain, resulting in kinase activation that is capable of transforming fibroblasts [42], [43]. The Nef–Hck interaction may be essential for macrophage-tropic HIV replication and AIDS pathogenesis [43], [44]. A full list of published HIV-1 nef interactions can be found at http://www.ncbi.nlm.nih.gov/RefSeq/HIVInteractions/nef.html.

In a healthy state, the brain is void of T- and B-lymphocytes and even when a patient is HIV-infected, little, if any, infiltration of immune cells into the brain other than monocytes and macrophages occurs. Other cells infected by HIV when it enters the brain are astrocytes [29], [45]. Neuronal cells are not infected with HIV, but cellular by-products produced by HIV-infected macrophages affect neurons, resulting in cognitive impairment. Potential HIV nef interactions are likely more restricted in the brain compartment than in tissues associated with the lymphatic system. With the task of crossing the blood-brain barrier (BBB) and limited cell types to infect, it seems feasible that only certain genotypes of HIV are capable of successfully infecting resident brain macrophages and, furthermore, only a subset of these genotypes have acquired the ability to induce the macrophage-associated HAD cascade within that particular cellular environment.

In this study we found some brain signatures that may affect protein folding and also a strong relationship between subtype B brain structures from patients with HAD and a subtype D consensus structure generated from 55 sequences originating from individual patients in Uganda, where infection with subtype D results in an 89% risk of developing HAD [7]. Interesting amino acid substitutions occurred in HAD viruses that may not have been obvious without mapping them to the 3D structures. The interaction between nef and SH3 is important for enhancement of virion infectivity and in MHC-I downregulation but not in downregulation of CD4 [46], [47], [48] and the only structure generated to date that shows the nef-SH3 interaction has an alanine at position 83, whereas all of our patients with brain infection had a glycine in this position. The ΔΔG of an A to a G is significant and could result in a gain-of-function for either SH3 binding or another unknown nef functional advantage. Glycine has more conformational freedom than any other amino acid in an unfolded protein; however, it has only one conformation in the folded protein and this alone could account for the brain specificity of the protein. The finding of disease-associated substitutions that are directly adjacent (positions 101 and 181) in the hydrophobic core structure of the internal anti-parallel beta sheet and a non-bonded cysteine residue in the flexible loop that is accessible for interactions is a new discovery. Because one of the core's primary functions is structural integrity, these substitutions could generate a final structure with modifications significant enough to make them unique when compared to other non-brain structures. Additionally, position 101 is within a domain required for chemokine induction [49]. Chemokines act as immuno-inflammatory mediators and have an important role in normal brain function [50]. Cells secrete nef [51], [52]; therefore, the cysteine mutation at position 162 could bind extracellular proteins. Alternatively, disulfide bridges are also formed in the endoplasmic reticulum, which is the first compartment of the secretory pathway [53]. This position also falls within a dileucine binding motif that can bind AP-2 and substitutions here could reduce both AP-2 binding and CD4 downregulation [54]. A glycosylation motif was eliminated in nef with the cysteine substitution. Although glycosylation of nef does not occur during HIV-infection, it is a factor considered in *in vitro* assays for vaccine development [55]. Additionally, since the one brain patient that did not have a cysteine at position 162 also did not have a glycosylation motif, it may be that the asparagine-asparagine-serine motif provides a structural rather than functional significance.

Comparison of closely related structures is useful in understanding how proteins accommodate slight alterations in amino acid sequences and the analysis of such changes is vital to the identification of protein attributes that could contribute to disease or treatment. Despite considerable amino acid variation between HIV subtypes, the finding that subtype B nef protein structures derived from brain tissues in two patients are more similar to Uganda subtype D structures than to the same patients' HIV nef structures derived from their non-brain tissues provides further evidence that a disease-associated virus exists. Although a considerable amount of brain derived sequence data exists within the public sector, relatively few HIV-nef sequences from well documented cases of HAD exist; we found only 112 sequences derived from HAD patients and only 41 of these were extracted from brain tissues from a total of 19 patients. Only two of the patients in these studies had multiple HIV-1 nef sequences from brain tissues available. Currently, the assessment of HAD is becoming more rigorous due to the use of the International HIV Dementia Scale [56]. These tests, in combination with the generation of large amounts of nef sequence data from patients with well-documented cases of HAD could permit the development of a system to identify HAD viruses through structural analysis. The development of a biomarker for HAD development could also aid in the development of drugs to target HAD-specific viruses.

## Supporting Information

Figure S1 **Phylogenetic Analysis.** All of the sequences used in the study were incorporated into the phylogenetic tree. Each patient and Uganda subtype A and D sequences are color-coded. The tree was calculated using the protein sequences and the PAM substitution matrix in MEGA 4.0. Bootstraping support values for the major branches of the tree are superimposed.
(TIF)
